# Novel diagnostic test for the detection of *Mycobacterium tuberculosis* and its resistance to rifampicin, isoniazid, and fluoroquinolones directly in sputum samples

**DOI:** 10.3389/ftubr.2025.1536600

**Published:** 2025-03-31

**Authors:** Gayathri Ramasubban, Joy Sarojini Michael, Richa Gupta, Kavyasri Channamaneni, Dhanabagyam Krishnan, Dev Chandran, Sven Hoffner, Pavankumar Asalapuram

**Affiliations:** ^1^EMPE Diagnostics Private Limited, Hyderabad, India; ^2^Department of Microbiology, Christian Medical College, Vellore, Tamil Nadu, India; ^3^Christian Medical College and Hospital, Vellore, Tamil Nadu, India; ^4^Department of Global Public Health, Karolinska Institutet (KI), Stockholm, Sweden; ^5^EMPE Diagnostics AB, Stockholm, Sweden

**Keywords:** pre-XDR-TB, fluoroquinolone, rapid diagnostics, rifampicin, isoniazid, MDR-TB

## Abstract

**Introduction:**

Rapid identification of tuberculosis (TB) and its drug resistance is crucial for starting effective treatment promptly and preventing the spread of resistant *Mycobacterium tuberculosis* (MTB) strains. Expanding the use of existing and new rapid molecular diagnostic tests is urgently needed to combat the rising threat of TB, multidrug-resistant TB (MDR-TB), and pre-extensively drug resistant TB (pre-XDR-TB). The *mfloDx*™ diagnostic platform was developed to provide efficient, accurate, and accessible TB diagnostics. This study evaluates the performance of the *mfloDx*™ pre-XDR-TB test for detecting TB and drug resistance against MGIT culture and drug susceptibility testing (DST).

**Methodology:**

We have evaluated the performance of *mfloDx*™ pre-XDR-TB test on 731 sputum samples received from a tertiary care center in India. This study compares the analytical and clinical efficiency of *mfloDx*™ pre-XDR-TB test against the MGIT culture for *M. tuberculosis* complex (MTC) and MGIT-DST for rifampicin (RIF), isoniazid (INH), and fluoroquinolone (FQ) resistance. The clinical sensitivity and specificity were calculated for TB and drug-resistance detection using MedCalc statistical software.

**Results:**

The *mfloDx*™ pre-XDR-TB test showed 86.2% of sensitivity and 82.0% of specificity for MTC detection against MGIT culture. For drug resistance detection, sensitivity and specificity were found to be 98.2% and 99.7% for RIF, 86.2% and 99.2% for INH, and 93.3% and 100% for FQ, respectively, while the Indeterminate rates were 1.1% for RIF, 2.0% for INH, and <1% for FQ. The *mfloDx*™ pre-XDR-TB test's high specificity minimized false positives, which is essential for preventing unnecessary treatments, while rapid results offered a significant advantage over conventional methods.

**Conclusion:**

The *mfloDx*™ pre-XDR-TB test efficiently provides a reliable, rapid and specific diagnostic results for TB and its drug resistance detection. While it shows potential for inclusion in the clinical diagnostic workflows, especially in high-burden areas, further optimization are required to enhance its sensitivity. Nonetheless, the test offers significant advantages for the prompt management of drug-resistant TB in resource-limited settings.

## Introduction

In 2023, tuberculosis (TB) re-emerged as the world's leading infectious disease killer, overtaking COVID-19 and remaining a major cause of death, especially for individuals with HIV and those affected by antimicrobial resistance. An estimated 10.8 million people globally fell ill with TB, with 400,000 cases developing multidrug-resistant or rifampicin-resistant TB (MDR/RR-TB) (1). Notably, 5.5% of those tested for rifampicin resistance were found to have MDR/RR-TB or even more resistant forms, underscoring the urgent need for effective interventions to curb TB and its drug-resistant strains (1).

Developing a rapid sputum-based diagnostic test for multidrug-resistant tuberculosis (MDR-TB) presents several challenges due to the complex biology of *Mycobacterium tuberculosis* (MTB), the heterogeneity of resistance-conferring mutations, and the variability in sputum samples. The thick, lipid-rich cell wall of TB complicates lysis and nucleic acid extraction, while low bacillary loads, particularly in HIV co-infected patients, hinder detection (2, 3). Molecular assays such as Xpert MTB/RIF Ultra (Cepheid) (4, 5) and Truenat MTB-RIF (Molbio) (6) offer rapid, automated detection of rifampicin resistance; however, their inability to detect isoniazid resistance and high costs limit widespread use in resource-limited settings. Line probe assays (e.g., GenoType MTBDRplus) provide expanded drug resistance profiling but require sophisticated laboratory infrastructure, restricting their applicability in high-burden regions (7). Phenotypic drug susceptibility tests (e.g., MGIT 960) remain the gold standard but require weeks for results, delaying treatment initiation (1). Thus, while substantial progress has been made, the ideal rapid, cost-effective, and comprehensive sputum-based MDR-TB diagnostic test remains an unmet need, necessitating further research and innovation.

To address these diagnostic shortcomings, the *mfloDx*™ diagnostic platform (EMPE Diagnostics AB, Sweden) has been developed, promising enhanced accuracy, speed, and accessibility. This platform combines two well-established technologies: (a) padlock probe-dependent rolling circle amplification (RCA) (8, 9), an isothermal nucleic acid amplification method, and (b) sensitive lateral flow nucleic acid biosensor chemistry for signal development readout. The *mfloDx*™ diagnostic platform has two products: the first product, *mfloDx*™ MDR-TB test (10), serves as a robust diagnostic tool for detecting TB and its resistance to RIF and INH, with a turnaround time of only 3 h. The second product, *mfloDx*™ pre-XDR-TB test is an advanced version of the MDR-TB test capable of detecting TB and resistance to RIF, INH, and FQ in a single test, also with a turnaround time of just 3 h, utilizing the existing infrastructure available in basic molecular biology laboratories.

In this report, we present preliminary evaluation data for the *mfloDx*™ pre-XDR-TB test using sputum samples, comparing the results against MGIT culture and drug susceptibility testing (DST) as the gold standards for TB detection and resistance detection for RIF, INH, and FQ.

## Methodology

The Institutional Ethics Committee (IRB No. 12191) approved the study protocol at CMC, Vellore. Seven hundred and thirty-one consecutive sputum samples received from the Department of Respiratory Medicine for routine Mycobacteriology analysis were processed and evaluated by MGIT culture and drug susceptibility tests (DST) and *mfloDx*™ pre-XDR-TB. The samples were collected in the out patient department (OPD) of Respiratory Medicine Unit of Christian Medical College, Hospital and transported to the Microbiology laboratory in the same campus immediately after collection. The majority of the samples were processed on the same day. In cases where immediate processing was not feasible, such as over weekends, samples were stored at 4°C and processed within 48 hours to maintain sample integrity. The samples consisted of a mix of early morning and spot samples. Since majority of samples were from OPD, only spot samples were collected. 69% of the samples were smear positive and 31% were smear negative. *mfloDx*™ pre-XDR-TB showed a sensitivity of 87% against smear (pooled samples). The results were not included in the manuscript, since the objective was to evaluate *mfloDx*™ pre-XDR-TB against MGIT culture.

Decontamination of 1 ml sputum was performed using the NALC-NaOH method (11). The decontaminated pellet was divided into two parts: the first part was used as inoculum for MGIT culture. The sediment from the second part of the decontaminated sample was resuspended in 200 μL of sample preparation buffer and heat lysed at 95°C for 20 min. Since the *mfloDx*™ pre-XDR-TB test does not require DNA extraction/purification, 5 μL of lysed supernatant was used for the detection of TB, wild-type or mutations in *rpoB* for RIF, *katG, inhA* for INH, *gyrA* for FQ. The mutations detected by the *mfloDx*™ pre-XDR-TB test are mentioned in Table 1.

**Table 1:** Mutations detected by the target genes in *mfloDx*™ pre-XDR-TB test.

**Gene**	**Codon**	**Mutation**	**Amino acid change**
*rpoB*	516	G/T	D516Y
	516	A/T	D516V
	526	C/G	H526D
	526	C/T	H526Y
	526	A/G	H526R
	531	C/T	S531L
	531	C/G	S531W
	533	T/C	L533P
*katG*	315	G/C	S315T
	315	G/A	S315N
*inhA*	−15	C>T	
*gyrA*	90	C/T	A90V
	94	G/A	D94N
	94	G/T	D94Y
	94	G/C	D94H
	94	A/G	D94G
	94	A/C	D94A

The protocol for performing the *mfloDx*™ pre-XDR-TB test is similar to the *mfloDx*™ MDR-TB (10) test. The *mfloDx*™ pre-XDR-TB test can be performed in any basic molecular biology laboratory with standard laboratory equipment and infrastructure such as a thermal cycler, heat block, microfuge, and vortex mixer. The test consists of 6 steps after heat lysis, namely pre-amplification, PLP capturing the specific targets, purification of ligated circles using magnetic beads, RCA of the circles, restriction and digestion of the amplified single-stranded concatemers, and finally, development of visual signals on the lateral flow cassettes. A detailed description of the thermal profile and isothermal amplification profile are mentioned in Table 2.

**Table 2:** Description of thermal/isothermal amplification profile for the various steps of *mfloDx*™ pre-XDR-TB testing.

**Steps**	**Thermal/isothermal amplification profile**	
Pre-amplification	37°C—5 min 95°C—5 min 95°C—10 sec 64°C—30 sec 72°C—10 sec	25 cycles
			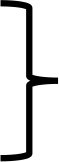
	95°C—10 sec 64°C—30 sec 72°C—30 sec 8°C—∞		15 cycles
				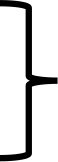
Ligation	94°C—1 min 56°C—5 min 8°C—∞		
Rolling circle amplification	37°C—20 min 65°C—1 min 8°C—∞	
Digestion	37°C—5 min 8°C—∞	

**Figure 1:**
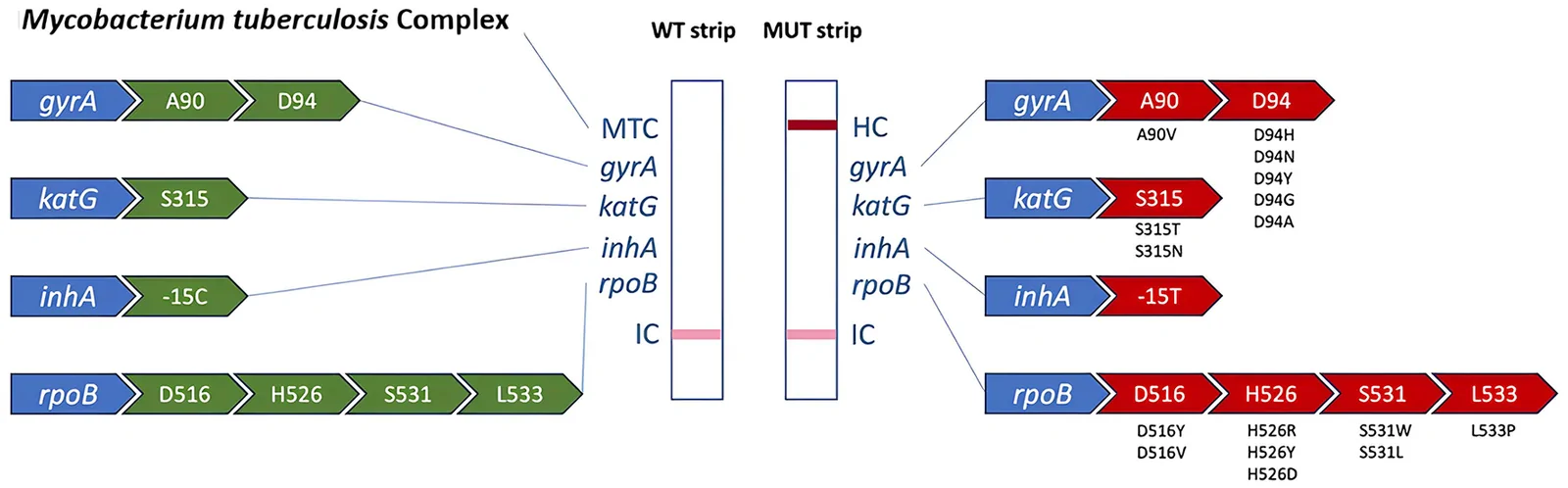
Overview of genomic loci represented by the respective bands of the *mfloDx*™ pre-XDR-TB test. On the left WT genotypes are represented, while on the right side we present the respective detected mutations.

As shown in Figure 1 the window on the left-hand side of the cassette, marked “WT,” indicates the presence of *M. tuberculosis* complex (MTC) and the detection of WT allele of the resistance-detecting codons. The right-hand side window of the cassette, marked “MUT,” shows the detection of mutations in the respective genes, while hybridization control to confirm the functionality of the visualization solution. The internal control band visible at the bottom of both the cassette windows shows the successful completion of all the steps of the *mfloDx*™ pre-XDR-TB test. If a band does not appear in either of the WT and MUT sides for *rpoB, katG, inhA*, and *gyrA*, it was interpreted as “indeterminate” to the respective drug. If a band appears on both the WT and MUT side for *rpoB, katG, inhA*, and *gyrA*, then it is interpreted as “heteroresistant” to the respective drug.

Drug susceptibility testing was performed using the BD Bactec MGIT system (Becton Dickinson Microbiology Systems, Cockeysville, MD) with MGIT liquid culture automation, following the manufacturer's instructions. Drug concentrations for rifampicin (RIF), isoniazid (INH), and fluoroquinolones (FQ) were set according to the Technical Manual for Drug Susceptibility Testing, WHO 2018 (12).

The clinical sensitivity and clinical specificity for the detection of MTC, RIF, INH and FQ resistance of the *mfloDx*™ pre-XDR-TB test were calculated using MedCalc statistical software (https://www.medcalc.org/calc/diagnostic_test.php) (13).

## Results

A total of 731 samples were included in this study. The MGIT culture examination showed that 542 samples (74.1%) were culture-positive, while 189 samples (25.9%) were culture-negative. The mfloDx™ pre-XDR-TB test detected TB in 68.5% of samples (501/731), while 31.5% (230/731) tested negative.

## Comparison of *mfloDx*™ pre-XDR-TB against MGIT culture for the detection of MTC

Among the 542 MGIT culture positive samples, 467 were MTC positive and 75 were MTC negative by the *mfloDx*™ pre-XDR-TB test. Out of the 189 MGIT culture negative samples, 34 were positive and 155 were negative for the detection of MTC by the *mfloDx*™ pre-XDR-TB test (Table 3). Thus the clinical sensitivity and specificity of the *mfloDx*™ pre-XDR-TB test against MGIT culture was found to be 86.2% and 82.0%, respectively.

**Table 3:** Comparison of the *mfloDx*™ pre-XDR-TB test against MGIT for MTC detection.

***N** =* **731**		**MGIT**	
		**Positive**	**Negative**
***mfloDx***™ **pre-XDR-TB**	**Positive**	467	34
	**Negative**	75	155
**Sensitivity (%)**		86.2	
**Specificity (%)**		82.0	

The following formula is used for the calculation of Sensitivity and Specificity.

Sensitivity = [TP / (TP + FN)] × 100.

Specificity = [TN / (FP + TN)] × 100.

TP, True positive; FN, False negative; TN, True negative; FP, False positive.

Among the 542 culture positive samples, 467 were positive by both MGIT and *mfloDx*™ pre-XDR-TB. MGIT DST results were available only for 454 samples and included in predicting the diagnostic accuracy calculation.

## Detection of RIF resistance

### *mfloDx*™ pre-XDR-TB vs. MGIT-DST

The results indicated that among the 56 samples identified as RIF resistant and 390 samples identified as RIF sensitive by both MGIT-DST and *mfloDx*™ pre-XDR-TB test. Additionally, there was one RIF-resistant sample by MGIT-DST, which was identified as sensitive by *mfloDx*™ pre-XDR-TB (Table 4). The *mfloDx*™ pre-XDR-TB test identified one sample as RIF resistant that was RIF-sensitive according to MGIT-DST. Furthermore, there were five samples classified as RIF indeterminate by the *mfloDx*™ pre-XDR-TB test, of which one was classified as RIF sensitive and four as RIF resistant by MGIT-DST (Table 4).

**Table 4:** Diagnostic accuracy of *mfloDx*™ pre-XDR-TB test using the MGIT phenotypic drug susceptibility test as a reference.

**Drug (*****n** =* **454)**		***R***	***S***	**Sensitivity (%, 95%CI)**	**Specificity (%, 95%CI)**	**PPV (%, 95%CI)**	**NPV (%, 95%CI)**	**Accuracy (%, 95%CI)**
RIF	R	56	1	98.2; 90.61–99.96	99.7; 98.59–99.99	98.2; 88.77–99.75	99.7; 98.25–99.96	99.5; 98.40–99.95
	S	1	391					
	ID	4	1	1.1				
INH	R	69	3	86.2; 76.73–92.93	99.2; 97.62–99.93	95.8; 88.13–98.62	97.0; 95.00–98.28	96.8; 94.78–98.27
	S	11	362					
	ID	6	3	2.0				
FQ	R	42	0	93.3; 81.73–98.60	100.0; 99.09–100.00	100.0; 91.59–100.00	99.3; 97.83–99.75	99.3; 98.06–99.86
	S	3	404					
	ID	0	2	<1%				
	HR	1	2	<1%				

RIF, Rifampicin; INH, Isoniazid; FQ, Fluoroquinolone; n, Number of samples; R, Resistant; S, Sensitive; CI, Confidence interval; PPV, Positive predictive value; NPV, Negative predictive value; ID, Indeterminate; HR, Hetero resistant.

## Detection of INH resistance

Among the 454 MGIT culture-positive samples, 69 samples were isoniazid-resistant and 362 samples were isoniazid-sensitive by both MGIT-DST and *mfloDx*™ pre-XDR-TB test (Table 4). Additionally, 11 isoniazid-resistant samples by MGIT-DST were identified as isoniazid sensitive, and 3 isoniazid-sensitive samples were resistant by *mfloDx*™ pre-XDR-TB The remaining 9 samples showed indeterminate results for isoniazid sensitivity testing by *mfloDx*™ pre-XDR-TB test. The results are detailed in Table 3. Excluding the indeterminate results, the sensitivity and specificity of the *mfloDx*™ pre-XDR-TB test were determined to be 86.2% and 99.2%, respectively, for INH resistance.

## Detection of FQ resistance

In this study, a total of 454 samples were analyzed using the MGIT-DST and *mfloDx*™ pre-XDR-TB test to assess FQ resistance. The results are summarized in Table 4.

The *mfloDx*™ pre-XDR-TB test identified 42 samples as FQ resistant and 404 as FQ sensitive, with no false positives reported in the FQ-sensitive group. Of the FQ-resistant samples, only 3 were identified as sensitive by the *mfloDx*™ pre-XDR-TB test. The FQ indeterminate and heteroresistance rate was <1%. Excluding the indeterminate results, the sensitivity and specificity of the *mfloDx*™ pre-XDR-TB test were determined to be 93.3% and 100%, respectively, for FQ resistance.

## Discussion

Several molecular diagnostic tests have been developed for the direct detection of TB and its drug resistance from clinical samples. However, each test has its advantages and limitations. The *mfloDx*™ pre-XDR-TB test can be performed in any basic molecular biology laboratory with standard laboratory equipment and infrastructure such as a thermal cycler, heat block, microfuge, and vortex mixer. In this study, we evaluated the performance of the *mfloDx*™ pre-XDR-TB test for detecting MTC and drug resistance to RIF, INH, and FQ. We compared its performance against MGIT-DST. The results demonstrated that the *mfloDx*™ pre-XDR-TB test offered several advantages but highlighted some limitations compared to the existing diagnostic landscape for tuberculosis (TB) and drug-resistance detection.

The *mfloDx*™ pre-XDR-TB test differs significantly from Xpert MTB/RIF Ultra (Cepheid) and Truenat MTB-RIF in multiple aspects, including the amplification method, target detection capabilities, and infrastructure requirements. Xpert MTB/RIF Ultra, a widely used automated PCR-based test, detects *M. tuberculosis* and rifampicin resistance in ~90 min. It utilizes real-time PCR with a nested design to enhance sensitivity, making it highly effective in diagnosing TB, even in some smear-negative cases. Truenat MTB-RIF, another real-time PCR-based test, is designed for decentralized settings and operates on a portable, battery-operated platform. While it enables rapid, sting for TB and rifampicin resistance in two tests, it does not extend to INH or FQ resistance detection. In contrast, *mfloDx*™ pre-XDR-TB, although requiring standard molecular biology equipment, allows for a broader scope of resistance detection, making it more informative for guiding treatment decisions in MDR-TB and detection of pre-XDR cases. While Xpert MTB/RIF Ultra and Truenat MTB-RIF provide rapid, user-friendly TB and rifampicin resistance detection, they cannot identify INH and FQ resistance. The *mfloDx*™ pre-XDR-TB test, with its RCA-based methodology, offers a broader resistance profile in a single assay, making it useful for identifying pre-XDR TB cases. The choice of assay depends on the clinical and infrastructural context, with Xpert MTB/RIF Ultra excelling in rapid TB detection, Truenat MTB-RIF enabling decentralized testing, and *mfloDx*™ pre-XDR-TB providing an expanded resistance detection capability in standard laboratory settings.

The clinical sensitivity and specificity of *mfloDx*™ pre-XDR-TB for the detection of TB compared to MGIT culture were 86.2% and 82.0%, respectively. The lower sensitivity against MGIT culture suggests that the *mfloDx*™ pre-XDR-TB test may miss a portion of TB-positive cases, particularly in low bacterial load samples. However*, mfloDx*™ still presents a valuable alternative with its near-perfect specificity, reducing the likelihood of false positives, which can lead to unnecessary treatment.

In terms of RIF resistance detection, the *mfloDx*™ pre-XDR-TB test performed strongly when compared with MGIT-DST. Against MGIT-DST, it achieved a sensitivity of 98.2% and specificity of 99.7%. The *mfloDx*™ pre-XDR-TB test's high specificity minimizes the risk of erroneous results in RIF resistance detection, a critical advantage in the management of drug-resistant TB, as overdiagnosis of RIF resistance could lead to unnecessary second-line treatments. Additionally, the indeterminate rate of 1.1% highlights a minor limitation in the assay, suggesting that a small proportion of results may require further testing or confirmation.

For INH resistance detection, the *mfloDx*™ pre-XDR-TB test exhibited a sensitivity of 86.2% and specificity of 99.2% when compared to MGIT-DST. These results demonstrate a strong capacity for detecting true INH-resistant cases while maintaining a high specificity. However, the 86.2% sensitivity indicates that a notable proportion of INH-resistant cases (11 samples) were missed by *mfloDx*™ pre-XDR-TB test. INH resistance in TB is often linked to multiple mutations in genes such as *katG, inhA, oxyR-ahpC* and *kasA* (14–16) and the variability in these resistance mechanisms may contribute to false-negative results. Published studies (7) on molecular diagnostics of INH resistance such as LPA have reported similarly high specificities for INH detection but often show higher sensitivity, particularly in settings with a high burden of resistance.

The 2% indeterminate rate for INH detection also indicates that while the test performs well overall, there is room for improvement in its ability to deliver conclusive results in all cases. This is particularly relevant in high-burden settings where rapid decision-making is critical, and indeterminate results may delay appropriate treatment initiation.

The *mfloDx*™ pre-XDR-TB test showed excellent performance in detecting FQ resistance, with a sensitivity of 93.3% and specificity of 100%. It has identified all but three FQ-resistant samples while reporting no false positives in FQ-sensitive samples. This high level of accuracy is crucial for pre-XDR-TB and XDR-TB management, as FQs are among the most important drugs in second-line TB treatment regimens. The test's performance in FQ detection surpasses many other molecular diagnostics, which have shown variability in their sensitivity to detect FQ resistance due to the complexity of mutations in the *gyrA* and *gyrB* genes. Additionally, the <1% heteroresistance rate further underscores its precision in identifying resistant populations within mixed infections.

## Advantages and limitations of the *mfloDx*™ pre-XDR-TB test

### Advantages

The *mfloDx*™ demonstrates high specificity, particularly against Xpert MTB/RIF, ensuring that false positives are minimized, which is essential to avoid unnecessary treatment. The high sensitivity and specificity for FQ resistance detection highlight its utility in identifying drug-resistant TB cases that require second-line treatments. Like other molecular assays, the *mfloDx*™ pre-XDR-TB test offers rapid results, which is a significant advantage over phenotypic methods like MGIT-DST, which can take weeks to yield results. In addition to detecting MTC, *mfloDx*™ pre-XDR-TB also screens for key drug resistances, making it a valuable tool in managing MDR-TB and pre-XDR-TB.

### Limitations

Like other molecular tests, the *mfloDx*™ pre-XDR-TB test has certain limitations. The prevalence and diversity of drug resistance mutations vary across MTBC lineages, which could influence the results, particularly in regions with high lineage diversity. There is a possibility that some resistance-conferring mutations may not be detected in certain samples. The *mfloDx*™ pre-XDR-TB test focuses on the most common mutations in *rpoB, katG, inhA*, and *gyrA*, which means rarer or alternative mutations may be missed. Future studies incorporating sequencing-based approaches could provide a more comprehensive understanding of drug resistance patterns. Additionally, the observed indeterminate rates for RIF (2%), INH (2%), and FQ (<1%) resistance indicate that, in some cases, results may not be definitive, necessitating repeat testing or confirmation using alternative methods.

## Conclusion

The *mfloDx*™ pre-XDR-TB test offers a valuable and rapid diagnostic tool for detecting TB and drug resistance, with several advantages in terms of specificity and fluoroquinolone resistance detection. While it shows slight limitations in sensitivity for detecting MTC, it remains a strong contender for integration into diagnostic workflows, particularly in settings with a high burden of drug-resistant TB. However, its performance in detecting RIF, INH, and FQ resistance could benefit from further optimization, and confirmatory testing may still be necessary in some cases.

## Data Availability

The original contributions presented in the study are included in the article/supplementary material, further inquiries can be directed to the corresponding author.
